# Spontaneous Mediastinal Emphysema

**DOI:** 10.7759/cureus.2369

**Published:** 2018-03-26

**Authors:** Devina Singh, Sanjana Kumar, Thor S Stead, Latha Ganti

**Affiliations:** 1 Medicine, University of Louisville; 2 Molecular and Cell Biology, University of California, Berkeley; 3 Student, Alpert Medical School of Brown University; 4 Clinical Sciences, University of Central Florida College of Medicine, Orlando, USA

**Keywords:** spontaneous, mediastinal, emphysema, hamman's syndrome, esophagus, chest cavity

## Abstract

The author presents a case of spontaneous mediastinal emphysema, also known as the Hamman’s syndrome. This case presentation highlights the common features of spontaneous mediastinal emphysema and reminds the clinician to have an index of suspicion for this diagnosis.

## Introduction

Spontaneous mediastinal emphysema (Hamman’s syndrome) is an uncommon medical condition. In the United States, it is estimated to have a prevalence ranging from one per 800 to one per 42,000 pediatric patients presenting to the hospital emergency department and approximately one in 30,000 emergency department referrals [1]. It most commonly occurs in the young males, with the average age of affected patients being 11 years, and it is associated with inhalational drug use. Though it is a benign and self-limiting condition, it often leads to a number of investigations because of the possibility of the misdiagnosis or rupture of any of the viscera. However, this condition rarely leads to any complications.

## Case presentation

A 15-year-old male was referred to our emergency department (ED) for the evaluation of the subcutaneous emphysema. The patient had presented with the gradual onset of dull resting chest pain lasting for almost an hour. The nature of the pain subsequently changed to sharp and radiated to his throat. He underwent a chest X-ray at an outside ED, which was remarkable for the subcutaneous emphysema. The laboratory evaluation revealed no abnormality. He was subsequently transferred to our emergency department without any further intervention. The patient was symptom-free upon arrival. He denied any history of trauma or prior such episodes. The physical examination revealed well-appearing male, alert, oriented, in no acute distress with palpable subcutaneous emphysema over the right trapezius muscle and neck. The vital signs were normal. A computed tomography (CT) scan of the chest was obtained.

**Figure 1:**
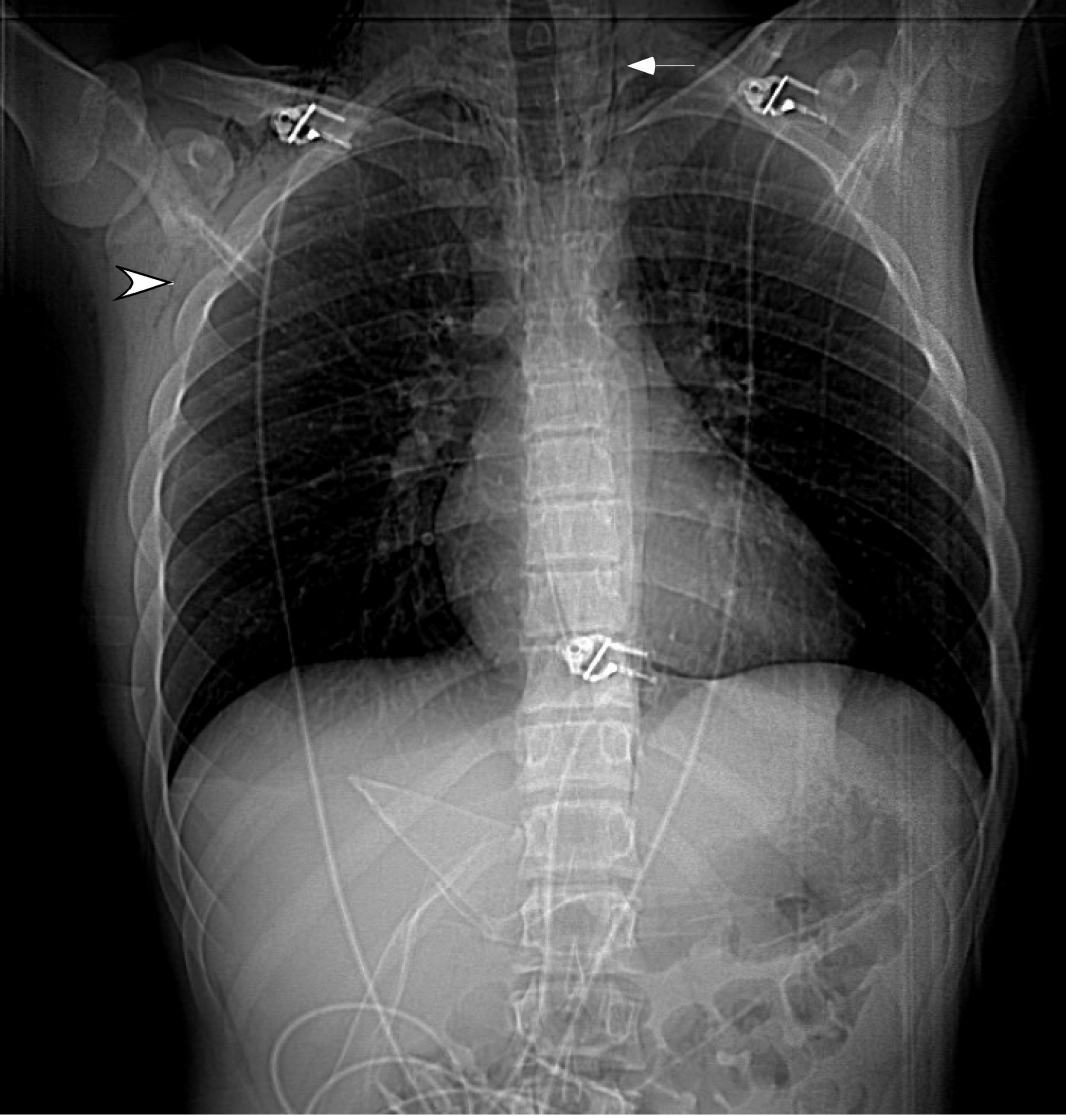
The scout's view depicting lucency along the left mediastinal border (arrow) indicating pneumomediastinum. Also, note the subcutaneous emphysema on the right side (arrowhead).

**Figure 2:**
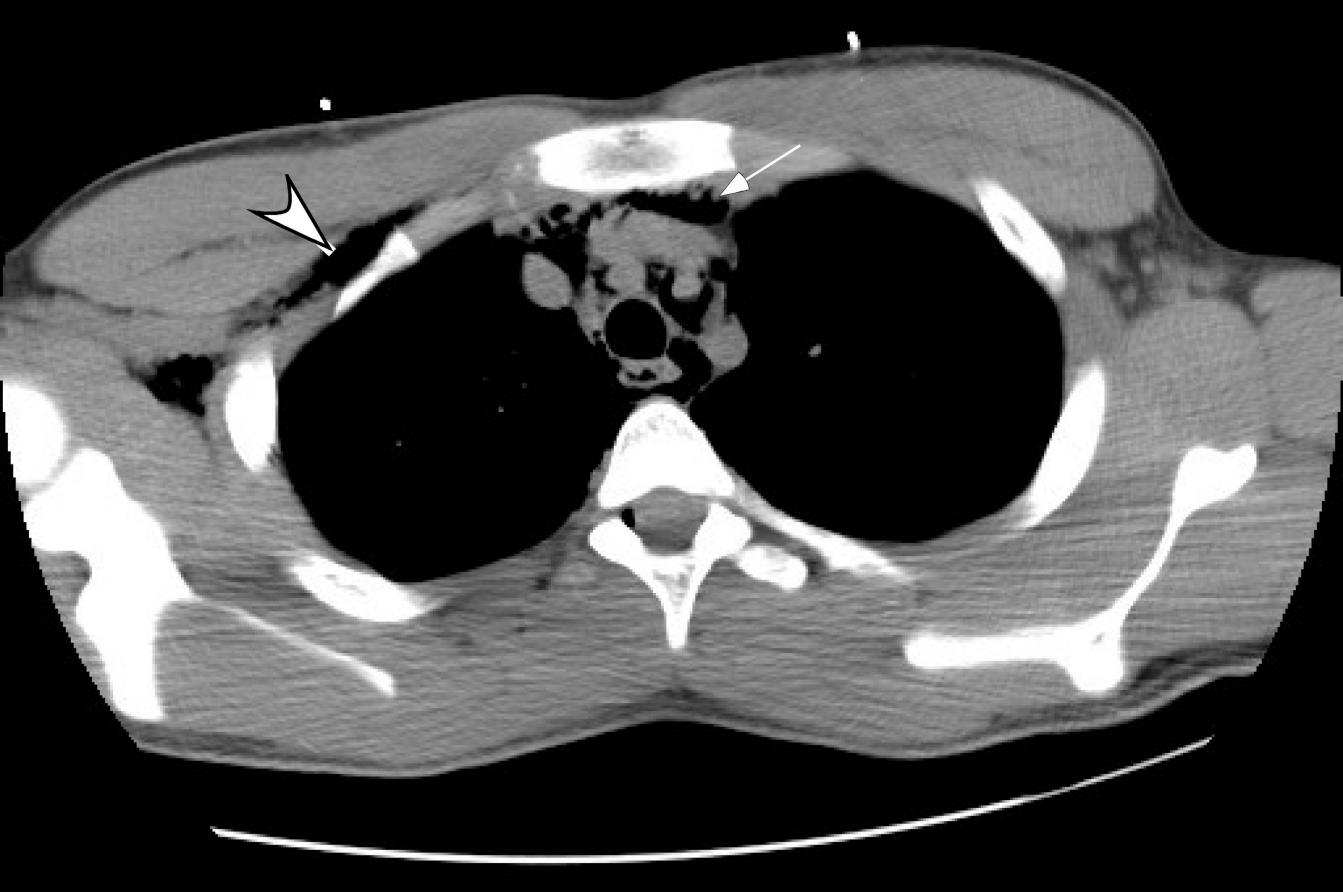
The computed tomography (CT) confirming the subcutaneous emphysema (arrowhead) and the extensive pneumomediastinum (arrow).

The chest CT confirmed the presence of subcutaneous emphysema as seen in Figure 1. There was additional evidence of extensive pneumomediastinum (Figure 2).

There was no pneumothorax, obvious esophageal, tracheolaryngeal perforation or fistula. The patient was observed for a few hours and subsequently dismissed. He did well during the follow-up and had an uneventful course.

## Discussion

The spontaneous mediastinal emphysema (Hamman’s syndrome) is a rare condition, which is usually benign and non-recurrent. It is known to be associated with transiently elevated intra-alveolar pressures. It can occur due to the blunt or penetrating trauma to the chest, forceful vomiting and medical procedures such as esophagoscopy and bronchoscopy [2]. The pathophysiology involves the alveolar rupture that leads to air distribution in the surrounding bronchovascular space and finally air dissemination from the pulmonary interstitium into the mediastinum [3]. In three retrospective analyzes, it has been noted that it commonly occurs in the young male patients and is associated with inhalational drug use [1-2, 4]. It has also been reported in the females with the absence of inhalational drug use [5].

The chest pain and dyspnea are the most prevalent presenting symptoms. The subcutaneous emphysema with or without the palpable crepitus is the most prevalent sign. Generally, the chest pain is retrosternal with the possible radiation to the neck or the back [3]. Hamman’s sign is a characteristic finding which is basically a precordial crunching note that is synchronous to the heartbeat [2]. It is present in more than half of the patients, though it was absent in this case. Most of the patients have normal vital signs and appear healthy [2].

The diagnostic testing involves the chest radiograph and if required, the computed tomography (CT) of the chest. A ‘ring sign’ can be appreciated on the chest X-ray which is caused by the presence of free air around the pulmonary artery or its branches [3]. The treatment is expectant with the pain management and the rest. The medical observation for a few hours or a few days is required depending on the patient symptoms and signs. The patients can be discharged safely as they do not develop any complications, nor do they have a recurrence [2]. In the event of any complication such as tension, pneumothorax or recurrence, the patients should be admitted to the hospital [3]. The follow-up chest radiographs in these patients may help in documenting the complete resolution of the condition.

## Conclusions

The spontaneous mediastinal emphysema is a relatively uncommon entity. Although the treatment is supportive, it is important to recognize, as it often leads to a number of investigations because of the possibility of a rupture of any of the viscera or the possibility of misdiagnosis. This case presentation highlights the common features of the spontaneous mediastinal emphysema and reminds the clinician to have an index of suspicion for this diagnosis.
